# A Markov Model Unveiling the Impact of Resmetirom on the Natural History of MASLD Patients: A Sistematic Review and Meta‐Analysis

**DOI:** 10.1111/liv.70056

**Published:** 2025-03-11

**Authors:** Grazia Pennisi, Gabriele Di Maria, Marco Enea, Marco Vaccaro, Ciro Celsa, Michela Antonucci, Giacinta Ciancimino, Carlo Ciccioli, Giuseppe Infantino, Claudia La Mantia, Adele Tulone, Vito Di Marco, Calogero Cammà, Salvatore Petta

**Affiliations:** ^1^ Section of Gastroenterology and Hepatology, Department of Health Promotion, Maternal and Child Health, Internal and Specialty Medicine of Excellence (PROMISE) University of Palermo Palermo Italy; ^2^ Department of Health Promotion, Maternal and Child Health, Internal and Specialty Medicine of Excellence (PROMISE) University of Palermo Palermo Italy; ^3^ Department of Economic, Business, and Statistical Sciences University of Palermo Palermo Italy; ^4^ Department of Biomedicine, Neurosciences, and Advanced Diagnostics (BIND) University of Palermo Palermo Italy

**Keywords:** cirrhosis, complications, fibrosis, Markov, MASLD, mortality, Resmetirom

## Abstract

**Background and Aim:**

The MAESTRO‐NASH phase 3 trial reported that a 52‐week treatment of Resmetirom is effective in improving fibrosis and metabolic dysfunction‐associated steatohepatitis (MASH) in patients with MASH and F2 or F3 fibrosis, while data on the impact on 5‐year and long‐term clinical outcomes are still lacking. We simulated the transition probabilities of disease progression in MASLD patients with F2 or F3 fibrosis and the effect of Resmetirom treatment on clinical outcomes.

**Methods:**

A meta‐analysis of literature data formed transition matrices for fibrosis stages and complications, defined as compensated (CC) and decompensated cirrhosis (DC), hepatocellular carcinoma (HCC) and mortality—liver‐related mortality (LR‐M), cardiovascular mortality (CV‐M) and extra‐hepatic cancer mortality (EHC‐M). Markov model was developed to depict the F2 and F3 fibrosis stage progression towards the complications and to evaluate the effect of Resmetirom treatment on the natural history of MASLD.

**Results:**

We estimated the 5‐year probability of Resmetirom‐treated and untreated MASLD patients with baseline F2 fibrosis of developing CC (5.16% vs. 6.82%, respectively), DC (0.25% vs. 0.3%, respectively), HCC (0.25% vs. 0.32%, respectively) and mortality (0.15% vs. 0.16% for LR‐M; 1.02% vs. 1.1% for CV‐M; 1.07% vs. 1.2% for EHC‐M, respectively). Similarly, we estimated the five‐year probability of Resmetirom‐treated and untreated MASLD patients with baseline F3 fibrosis of developing CC (17.12% vs. 21.34%, respectively), DC(1.1% vs. 1.47%, respectively), HCC (1.21% vs. 1.73%, respectively) and mortality (0.59% vs. 0.91% for LR‐M, 1.92% vs. 2.14% for CV‐M and 1.04% vs. 1.14% for EHC‐M, respectively). Life Years Gained (LYG) of Resmetirom‐treated patients were 0.45 and 0.63 in MASLD patients with F2 and F3 fibrosis, respectively, and the model was sensitive to changes in Resmetirom efficacy and transition probabilities.

**Conclusions:**

Resmetirom decreases the 5‐year and lifetime Markov‐model estimated risk of CC, DC, HCC and liver‐related mortality in patients with MASLD and F2 or F3 fibrosis.

**Summary**

- Data on the impact of Resmetirom on 5‐year and long‐term clinical outcomes are still lacking.
- In our simulated Markov model, individuals undergoing ResmetIrom treatment, in contrast to those untreated, have a lower estimated five‐years and lifetime risk of progressing to both compensated and decompensated cirrhosis, a reduced estimated susceptibility to HCC development, an overall lowered risk of liver‐related mortality and an increase in life year gained.
- In our model the Resmetirom‐regimen administration exerts a higher global benefit on clinical outcomes in MASLD with F3 fibrosis than those with F2.

**Abbreviations**
CC: compensated cirrhosis
CV‐M: cardiovascular mortality
DC: decompensated cirrhosis
EHC‐M: extra‐hepatic cancer mortality
FDA: food and drug administration
HCC: hepatocellular carcinoma
LR‐M: liver‐related mortality
LT: liver transplantation
MASLD: Metabolic‐dysfunction associated steatotic liver disease

## 1 Introduction

Metabolic dysfunction‐associated steatotic liver disease (MASLD) is swiftly becoming an emerging global health concern, placing a heavy burden on public health economics worldwide.

The most recent forecasts showed a continuous growth in MASLD diagnosis, following the rising trajectories of Diabetes and Obesity [1]. This upward trend predicts not only an increase in complications associated with liver cirrhosis, including liver decompensation and hepatocellular carcinoma (HCC) but also a worrisome increase in MASLD‐related all‐cause mortality—such as liver‐related mortality (LR‐M), cardiovascular mortality (CV‐M) and extra‐hepatic cancer mortality (EHC‐M)— [2].

The natural course of the disease has been widely explored but it remains not completely understood because of heterogeneity among studies. In this complex clinical setting, liver fibrosis represents the keystone of MASLD progression towards liver‐related complications and all‐cause mortality, albeit its significant variability among populations and individuals [3].

There are no yet approved drugs tailored specifically for MASLD and MASH, and among the therapeutic pipeline in development, Resmetirom, an agonist of thyroid hormone receptor (THR)‐β, has stepped into the limelight for its promising efficacy [4]. Preliminary analysis from the phase 3 trial MAESTRO‐NASH showed the efficacy of a 100 mg dose of Resmetirom on both MASH resolution without fibrosis impairment and ≥ 1 stage improvement in fibrosis without MASH impairment, compared to placebo, after a 52‐week regimen, earning the Food and Drug Administration (FDA) conditional approval for MASLD patients with F2–F3 fibrosis [5]. However, while waiting for long‐term results, data on Resmetirom's impact on the MASLD clinical course of those individuals is limited to a one‐year timeframe, resulting in a lack of comprehensive insight regarding its effects over longer periods.

The aim of this study is to model the potential long‐term impact of Resmetirom treatment on hard clinical outcomes in patients with F2–F3 fibrosis related to MASLD.

## 2 Methods

### 2.1 Markov Model Development

Data were extracted from the literature as part of a systematic review of 40 studies [6] on modelling the progression of disease in MASLD. Twelve studies were selected based on their relevance and alignment with the inclusion criteria, specifically those reporting transition probabilities or transition matrices between different disease stages and having similar patient recruitment criteria to minimise heterogeneity among studies included in the meta‐analysis. Each of these selected studies provided transition probabilities between disease stages (F1‐F2‐F3, compensated cirrhosis, and decompensated cirrhosis [DC]) as well as transitions towards MASLD‐related complications. In particular, studies reporting transition probabilities for liver transplantation, HCC, LR‐M, extrahepatic cancer‐related mortality (EHC‐M), and CV‐M were considered.

The selected studies include Younossi et al. [7], Tampi et al. [8], Adams et al. [9], Alswat et al. [10], Estes et al. [1, 11, 12], Goossens et al. [13], Swain et al. [14], Phisalprapa et al. [15], and Pearson et al. [16]. Neither progression nor regression probabilities were directly extracted from these studies. Instead, only the reported transition probabilities or transition matrices were collected and subjected to a meta‐analysis of proportions using a random‐effects model. This meta‐analysis synthesised the extracted data to generate a single, comprehensive transition matrix, which served as the basis for the Markov model.

A semi‐Markov with recurrent states model was performed (with the possibility of regressing states in addition to progression) following the scheme depicted in Figure S1. The model was implemented based on the transition probabilities derived from the matrix obtained through meta‐analysis, and two different starting stages of the disease were considered: the first one from stage F2, while the second from F3. In both models, 10,000 patients were simulated using pseudo‐random Monte Carlo extractions. Both Markov models simulate the potential lifelong progression of the disease for a hypothetical patient, with transitions occurring in annual cycles. Treatment is assumed to be administered during the first cycle (year), while outcomes are evaluated at 5 years post‐treatment and over the patient's lifetime. All analyses were performed using the R software (version 4.2.3; R Foundation for Statistical Computing (Vienna, Austria)).

### 2.2 Resmetirom Intervention

The clinical trial conducted by Harrison et al. [5], known as MAESTRO‐NASH, reached Phase 3. The study design included two treatment arms with Resmetiron at different dosages (80 and 100 mg) and a control arm with a placebo. The results indicated that the most effective dosage of Resmetiron was 100 mg per day, with a 26% increase in the probability of regression of at least one stage of fibrosis without MASH impairment in the first year of treatment. Given these findings, additional Markov models were developed following the same criteria as the natural history models, with the transition probabilities of the matrix adjusted based on the results of the Harrison et al. [5] study. In these models, the drug is assumed to be administered for 1 year, simulating disease progression and/or regression, the occurrence of complications, and mortality over a 5‐year and lifetime horizon.

### 2.3 Sensitivity Analysis

The developed models were compared using the Life Years Gained (LYG) indicator. Two sensitivity analysis strategies were implemented. The first is the Univariate Deterministic, where each cell in the transition matrix and treatment efficacy are varied by ±20%, and a Markov model is estimated for each matrix. The second is the Multivariate Probabilistic: it follows the same principle as the former, with the difference that each cell is varied using a value drawn from a normal distribution, with the mean given by the transition probability and the variance given by its standard deviation (or drawn from a uniform distribution between 0 and 1).

## 3 Results

### 3.1 The Markov Chain Models Simulate MASLD Patients' Journeys

First, we performed a meta‐analysis of those transition probabilities from which transition matrices have been obtained (Table S1), and then, we developed a *de novo* Markov model with liver disease‐specific state transition probabilities.

**FIGURE 1:**
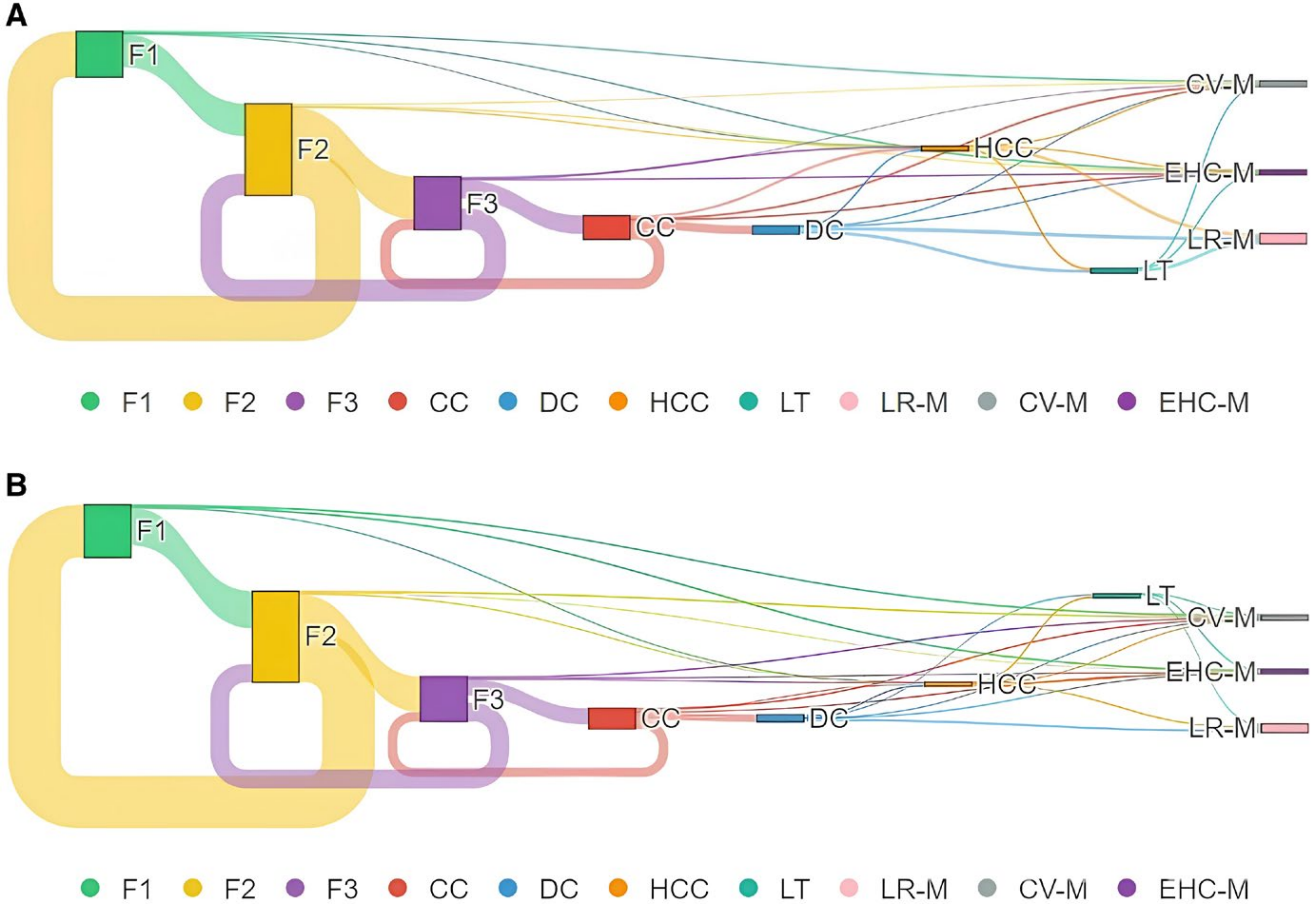
Sankey diagrams depicting the lifetime natural course of MASLD in untreated (A) and treated with Resmetirom (B) patients with F2 fibrosis. The width of the transitions' arrows is proportional to the number of patients placed in each specific stage. CC, compensated cirrhosis; CV‐M, Cardiovascular Mortality; DC, decompensated cirrhosis; EHC‐M, Extra‐Hepatic Cancer Mortality; HCC, hepatocellular carcinoma; LR‐M, liver‐related mortality; LT, liver transplantation; MASLD, metabolic‐dysfunction associated steatotic liver disease.

**FIGURE 2:**
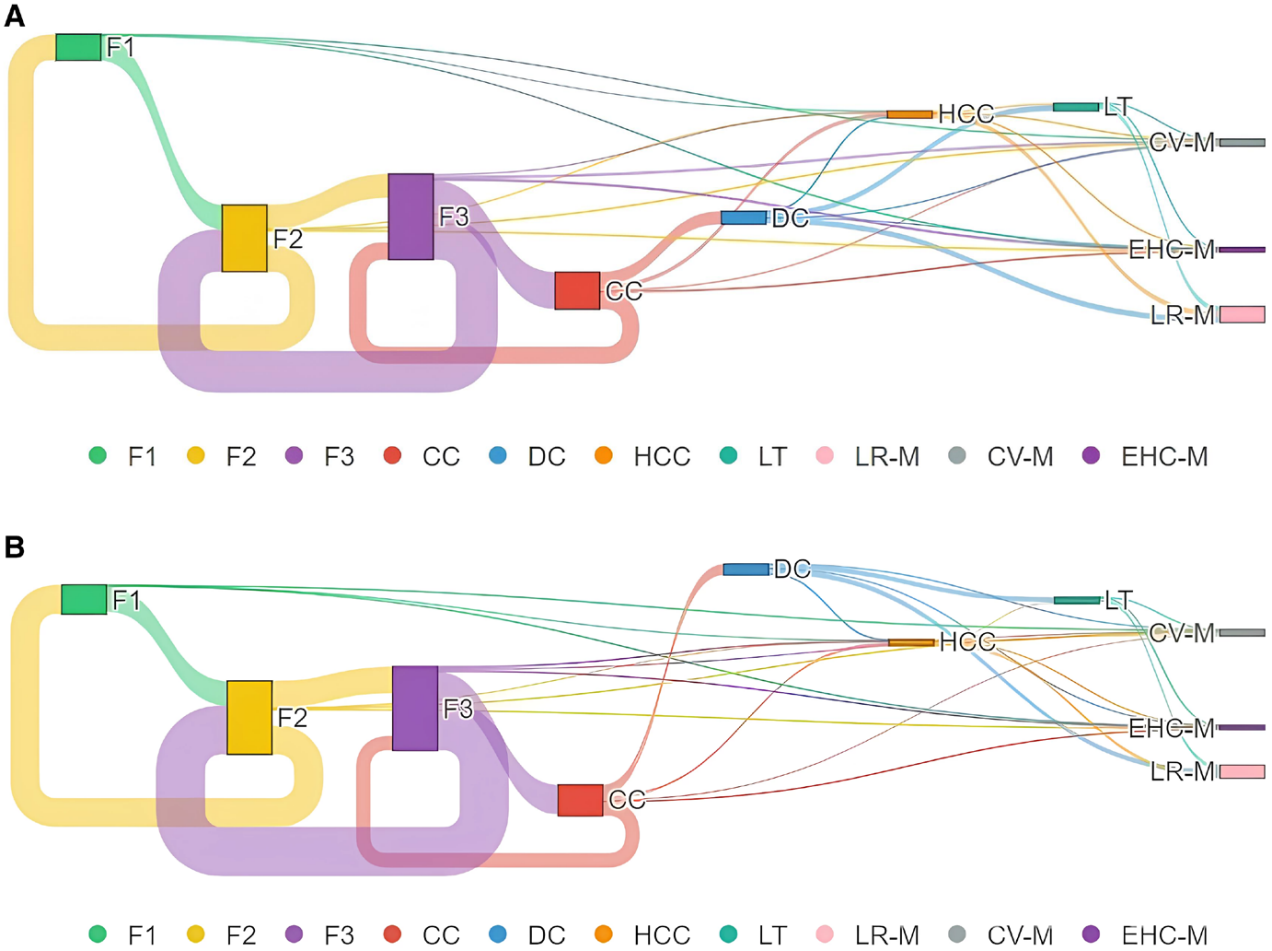
Sankey diagrams depicting the lifetime natural course of MASLD in untreated (A) and treated with Resmetirom (B) patients with F3 fibrosis. The width of the transition arrows is proportional to the number of patients placed in every specific stage. CC, compensated cirrhosis; CV‐M, Cardiovascular Mortality; DC, decompensated cirrhosis; EHC‐M, Extra‐Hepatic Cancer Mortality; HCC, hepatocellular carcinoma; LR‐M, liver‐related mortality; LT, liver transplantation; MASLD, metabolic‐dysfunction associated steatotic liver disease.

The model allowed for transitions backwards and forwards among fibrosis stages, CC, DC, HCC and death (LR‐M, CV‐M, EHC‐M), as reported in Table S1. The Sankey diagrams in Figures 1A and 2A (*click here for the interactive charts*) depict the flow of the state transition journey of untreated MASLD patients with F2 and F3 fibrosis, respectively.

### 3.2 The Estimated Effect of Resmetirom's Intervention on MASLD Natural History

We used a Markov model to estimate Resmetirom's effect over 5‐year and lifetime periods on the transition between fibrosis stages, on the occurrence of hepatic complications, and on all causes of mortality in patients with MASLD with F2 and F3 fibrosis. LYG were also calculated.

The Sankey diagrams recapitulate the natural history of the Resmetirom‐treated (Figure 1B) and the untreated (Figure 1A) MASLD with F2 fibrosis. More in detail, the 5‐year and lifetime probabilities of individuals with baseline F2 fibrosis MASLD developing CC were lower after Resmetirom intervention (5.16% and 40.98%, respectively) than in untreated patients (6.82% and 43.1%, respectively) (Table 1). Similarly, the 5‐year and lifetime likelihoods of DC and HCC were confirmed lower in Resmetirom‐treated (0.25% and 13.51% for DC and 0.25% and 7.82% for HCC, respectively) than in untreated patients (0.3% and 14.35% for DC and 0.32% and 8.52% for HCC, respectively) (Table 1). The estimated 5‐year and lifetime probabilities of both LR‐M and CV‐M resulted lower in Resmetirom‐treated subjects with F2 fibrosis (0.15% and 16.27% for LR‐M, respectively; and 1.02% and 9.8% for CV‐M, respectively) than those untreated (0.16% and 17.8% for LR‐M, respectively; and 1.1% and 10.12% for CV‐M, respectively). When we looked at EHC‐M, the 5‐year probability was lower in Resmetirom‐treated than in the untreated group (1.07% vs. 1.2%, respectively); conversely, the lifetime estimated EHC‐M was slightly higher in Resmetirom‐treated than in the untreated group (7.98% vs. 7.88%, respectively) (Table 1). Overall, the LYG of Resmetirom‐treated MASLD patients with F2 fibrosis was 0.45 years.

**TABLE 1:** Simulated 5‐year and lifetime rates of developing events according to baseline MASLD fibrosis stage F2 or F3 with or without one‐year Resmetirom administration.

		Simulated 5‐years rates		Simulated lifetime rates	
Fibrosis stage	Events	Natural history	One‐year resmetirom intervention	Natural history	One‐year resmetirom intervention
F2	CC	6.82	5.16	43.1	40.98
	DC	0.3	0.25	14.35	13.51
	HCC	0.32	0.25	8.52	7.82
	LR‐M	0.16	0.15	17.8	16.27
	CV‐M	1.1	1.02	10.12	9.8
	EHC‐M	1.2	1.07	7.88	7.98
F3	CC	21.34	17.12	56.55	52.68
	DC	1.47	1.1	20.47	18.85
	HCC	1.73	1.21	11.62	10.42
	LR‐M	0.91	0.59	25.5	23.01
	CV‐M	2.14	1.92	11.31	11.37
	EHC‐M	1.14	1.04	7.18	7.36

*Note:* Rates are given as %.

Abbreviations: CC, compensated cirrhosis; CV‐M, cardiovascular mortality; DC, decompensated cirrhosis; EHC‐M, extra‐hepatic cancer mortality; HCC, hepatocellular carcinoma; LR‐M, liver‐related mortality.

The Sankey diagrams in Figure 2A,B summarise the natural history of the MASLD patients with F3 fibrosis in the Resmetirom‐treated and untreated groups, respectively.

The 5‐year and lifetime probabilities of individuals with baseline F3 fibrosis MASLD of developing CC were lower after Resmetirom intervention (17.12% and 52.68%, respectively) than in untreated patients (21.34% and 56.55%, respectively) (Table 1). Similarly, the 5‐year and lifetime likelihoods of DC and HCC were confirmed to be lower in Resmetirom‐treated (1.1% and 18.85% for DC and 1.21% and 10.42% for HCC, respectively) than in untreated patients (1.47% and 20.47% for DC and 1.73% and 11.62% for HCC, respectively) (Table 1). The estimated 5‐year and lifetime probabilities of LR‐M, as expected, were lower in Resmetirom‐treated (0.59% and 23.01%, respectively) than those untreated (0.91% and 25.5%, respectively). When we looked at CV‐M and EHC‐M, the 5‐year probability was lower in Resmetirom‐treated than in the untreated group (1.92% vs. 2.14% for CV‐M and 1.04% vs. 1.14% for EHC‐M); conversely, both lifetime estimated CV‐M and EHC‐M resulted slightly higher in Resmetirom‐treated than in the untreated group (11.37% vs. 11.315 for CV‐M, and 7.36% and 11.37% for EHC‐M) (Table 1). Overall, the LYG of Resmetirom‐treated MASLD patients with F3 fibrosis was 0.63 years.

### 3.3 Sensitivity Analysis

**FIGURE 3:**
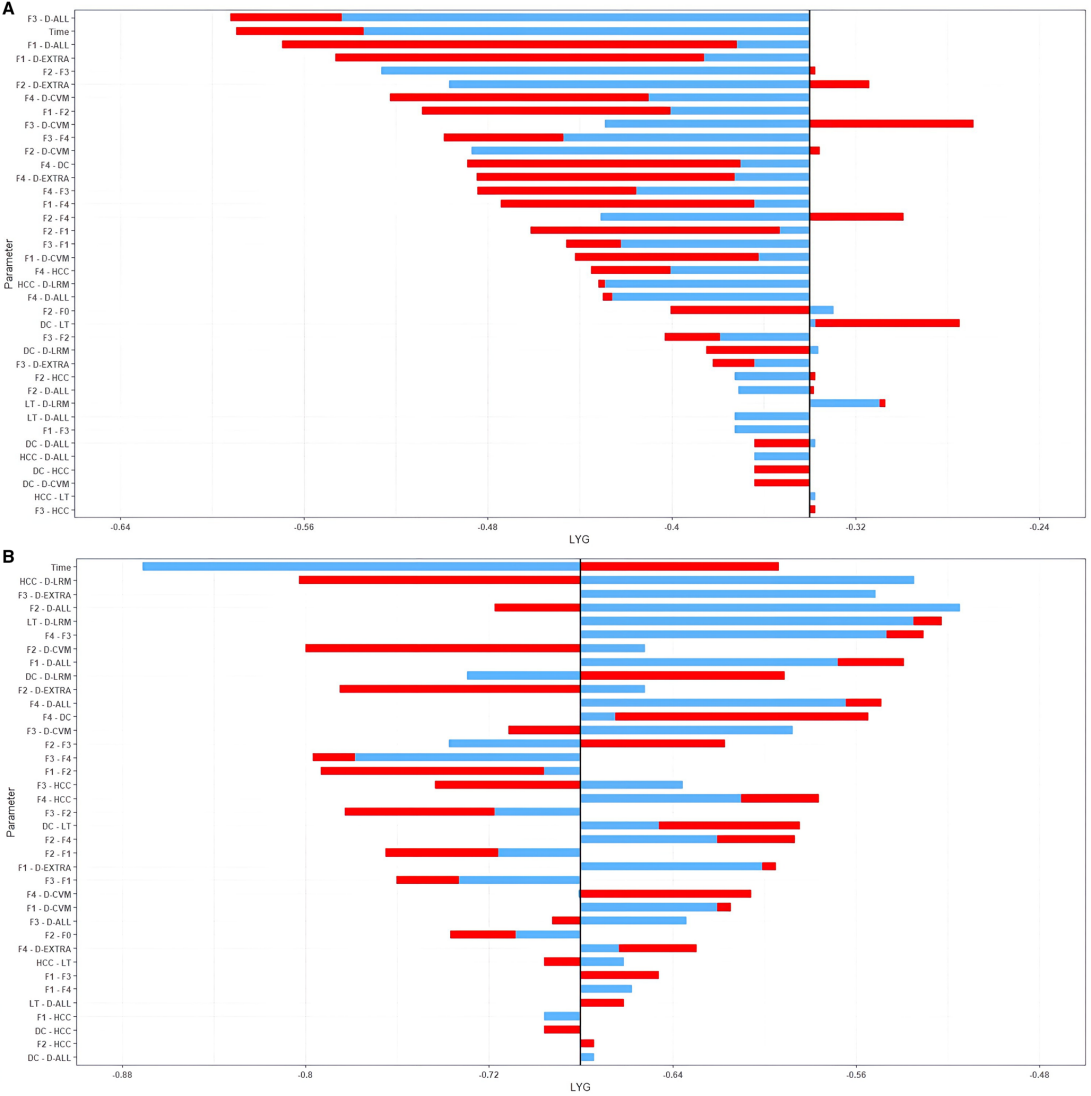
Tornado Plot of the Univariate Deterministic Sensitivity Analysis on F2 (A) and F3 (B) fibrosis MASLD patients showing the estimated efficacy of Resmetirom at the variation of Resmetirom efficacy and transition probabilities by +20% (red bars) and −20% (blue bars). CC, compensated cirrhosis; CV‐M, Cardiovascular Mortality; DC, decompensated cirrhosis; EHC‐M, Extra‐Hepatic Cancer Mortality; HCC, hepatocellular carcinoma; LR‐M, liver‐related mortality; LT, liver transplantation; MASLD, metabolic‐dysfunction associated steatotic liver disease.

Two sensitivity analysis strategies evaluated the Markov models' performance (Table S2). The Tornado Plots in Figure 3 illustrate the Univariate Deterministic Sensitivity Analysis on F2 and F3 fibrosis MASLD patients and show that the models are sensitive to variations in the efficacy of Resmetirom (±20% of fibrosis improvement) and to variations in transition probabilities of cirrhosis development, liver‐related complications and death by ±20%. Figure S2 illustrates the normal and uniform Multivariate Probabilistic Sensitivity Analysis.

## 4 Discussion

This study—based on a Markov chain model—was designed to depict and unveil insights into the 5‐year and lifetime impact of Resmetirom treatment on the MASLD natural course of patients with F2 and F3 fibrosis. It reveals that individuals undergoing treatment, in contrast to those untreated, have a lower estimated 5‐year and lifetime risk of progressing to both CC and DC, a reduced estimated susceptibility to HCC development, an overall lowered risk of LR‐M and an increase in LYG. Notably, as expected, the Resmetirom‐regimen administration exerts a higher global benefit on clinical outcomes in MASLD with F3 fibrosis than on those with F2.

MASLD lacks treatments, but Resmetirom shows promise and is gaining conditional FDA approval for F2 and F3 fibrosis. The MAESTRO‐NASH phase III trial [5] confirms histological efficacy at 52 weeks, with long‐term clinical outcomes pending. Our study, based on the construction of a Markov model, showed for the first time that the five‐year estimated probability of developing CC, DC, and HCC is lower in subjects with F2 and F3 fibrosis treated with Resmetirom compared to untreated ones. Consistent with the reduction in disease progression and occurrence of liver‐related events, our Markov models also demonstrated a 5‐year decrease in estimated risk of LR‐M in MASLD individuals with F2 and F3 fibrosis treated with Resmetirom compared to those untreated. Similarly, when we looked at a long‐term frame, the estimated lifetime risk of developing CC, DC and HCC and of experiencing LR‐M resulted in lower rates in both F2 and F3 MASLD patient groups treated with Resmetirom compared to untreated ones. Noteworthy, in both the 5‐year and long‐term timeframes, Resmetirom resulted overall better in patients with MASLD and fibrosis F3 compared to the F2 ones, pinpointing the patients' setting that could experience the greatest benefit from this therapeutic approach.

In the MAESTRO‐NASH phase 3 trial, Resmetirom achieved both FDA‐designated outcomes: resolution of NASH without fibrosis impairment and improvement in fibrosis by at least one stage without NASH impairment. Literature extensively documented that MASH accelerates the progression of fibrosis compared to a simple fatty liver, and longitudinal data further support that the severity of liver fibrosis is a crucial determinant of liver‐related events in MASLD subjects [17, 18, 19, 20]. Given this scenario, the effectiveness of Resmetirom on histological outcomes accounts for the reduction of disease progression and liver‐related events as estimated in our Markov model.

Focusing on extra‐hepatic death, MASLD patients with F2 and F3 fibrosis treated with Resmetirom have a reduced 5‐year estimated risk of CV‐M and EHC‐M compared to those untreated. Although Resmetirom selectively targets the liver‐specific THR‐β, exerting its action directly on it, it might indirectly influence other tissues and organs through the metabolic benefits of thyroid hormone mediated by the liver, including the reduction of excess hepatic fat and atherogenic lipids [21]. Expanding the timeframe, the estimated lifetime risk of EHC‐M in MASLD individuals with F2 fibrosis treated with Resmetirom is slightly higher than in those untreated, while Resmetirom‐treated MASLD individuals with F3 fibrosis had a slight increase in the estimated risk of both CV‐M and EHC‐M. The Resmetirom‐induced estimated low risk of advanced liver disease and its complications, including decompensation, HCC, and consequently LR‐M, could competitively slightly increase the risk of CV‐M and EHC‐M. Anyway, overall, we observed that Resmetirom treatment produced LYG of 0.45 and 0.63 years in patients with baseline F2 or F3 fibrosis, respectively.

Our study also showed that the model we built was sensitive to variations in Resmetirom efficacy and changes in transition probabilities to cirrhosis development, occurrence of liver‐related events and mortality. These data highlight as baseline characteristics of treated populations could strongly affect the impact of the therapy on clinical outcomes, demanding data for a personalised medicine approach.

Although our study is based on the simulation of Resmetirom administration over 1 year, the demonstrated benefits of Resmetirom make the long‐term effects observed in our study both biologically and clinically plausible. Resmetirom promotes mitochondrial fatty acid oxidation and suppresses lipogenesis, reducing liver fat accumulation and oxidative stress, which are key drivers of fibrosis, MAFLD and disease progression [22]. By decreasing steatosis and inflammation, it limits hepatic stellate cell activation, slowing or potentially reversing fibrosis and reducing the risk of cirrhosis and HCC. Chronic inflammation and fibrosis contribute to HCC development, but Resmetirom may lower HCC risk by mitigating these factors and regulating hepatocyte proliferation and apoptosis [23]. While it does not affect body weight, glucose or insulin resistance, Resmetirom significantly reduces LDL cholesterol, triglycerides and lipoprotein(a), which may lower cardiovascular events by 16%–21% [24]. Further studies, including the full MAESTRO program, are needed to confirm its long‐term benefits for liver complications and extra‐hepatic mortality.

From a clinical perspective, the results of our Markov model chains corroborate the already established short‐term efficacy of Resmetirom on histological outcomes and simulate how long‐term treatment with Resmetirom could bring about a paradigm shift in the natural history of MASLD patients with F2 and F3 fibrosis. The Resmetirom administration confers a comprehensive benefit on liver disease by reducing the estimated risk of cirrhosis and its complications, as well as LR‐M, over an unexplored and extended temporal horizon.

Delving into clinical practice, the potential introduction of Resmetirom to the market could open new therapeutic perspectives in the coming years. Currently, lifestyle interventions are key in MASLD/MASH management, improving insulin resistance, promoting weight loss, and reducing liver fat [25, 26, 27]. Moreover, lifestyle changes are cost‐effective and widely accessible. However, long‐term adherence can be challenging for many patients. In these cases, Resmetirom offers a valuable addition, enhancing liver protection and fibrosis reduction. It can be used alongside lifestyle changes or as an alternative when they are not feasible or effective. Their combination may provide the best outcomes—lifestyle improvements in weight and insulin sensitivity, while Resmetirom adds cardiovascular protection by lowering atherogenic lipoproteins [28].

Despite these optimistic results, one of the major shortcomings of our Markov models is the simulation of the estimated risk of clinical outcomes based on 52‐week Resmetirom administration results from the phase 3 clinical trial. Currently, there is no data on the duration of Resmetirom's efficacy on histological outcomes and thus on the maintenance of long‐term reduction of disease progression, which would also impact clinical outcomes. A study conducted by Ratziu et al. [29] highlighted that histological improvement after Rosiglitazone administration in MASLD patients occurred only after the first‐year treatment, with no further benefits from the second year onwards. Important additional future extensions of this work with trial‐derived and real‐life data are mandatory to validate the efficiency of this model prediction. On the other hand, our models could underestimate the positive effect of Resmetirom treatment on the natural history of MASLD patients with F2 or F3 fibrosis: it is, in fact, possible that Resmetirom (1) could maintain/increase its efficacy in 1‐year responder patients, and (2) could also lead to histological improvement in 1‐year nonresponder patients. Furthermore, the lack of individual data and the absence of validation on key assumptions compound the previously mentioned drawback.

Following the previous limitation, another key issue is that the long‐term benefits of the Resmetirom‐treated population seem somewhat limited compared to the untreated one, raising concerns about its cost‐effectiveness over an extended period. However, this could arise from the results based on only 1 year of treatment, which might not be sufficient to fully capture Resmetirom's hepatoprotective and antifibrotic effects, as well as its potential broader benefits on cardiovascular health. A longer treatment duration could offer further insights into whether these initial benefits are sustained and whether they would ultimately justify the cost of the treatment in clinical practice.

Another significant limitation arises from the construction of the model itself and the transition probabilities used in its development. A meta‐analysis of transition probabilities from recent published studies was performed to obtain updated transition matrices underlying the Markov model chains, addressing this issue. After all, our Markov models address an area of urgent and still unmet clinical need, in which the long‐term effectiveness data of drugs under investigation for MASLD patients are lacking. We believe that our simulation models could provide an anticipated evaluation of liver outcome over an extended (5‐years and lifetime) timeframe and could empower the information about the whole spectrum of complications (included the extra‐hepatic ones) in MASLD patients undergoing future clinical trials.

In conclusion, Resmetirom treatment in MASLD patients with F2 or F3 fibrosis reduces the 5‐year and lifetime Markov model estimated risk of progression to compensated and DC and of HCC occurrence, and lowers the risk of LR‐M, overall increasing LYG. Trial‐derived and real‐life studies are needed to corroborate this Markov model simulated scenario and to confirm that Resmetirom might rewrite the natural history of patients with MASLD and F2–F3 fibrosis.

## Author Contributions

G.P., G.D.M., M.E., M.V., C.C., M.A., G.C., C.C., G.I., C.L.M., A.T., V.D.M., C.C., S.P. had full control of the study design, data analysis, interpretation and preparation of the article. All authors were involved in planning the analyses and drafting the article. The final draft of the article was approved by all the authors.

## Disclosure

The authors have nothing to report.

## Ethics Statement

The authors have nothing to report.

## Conflicts of Interest

S.P. has acted as speaker and/or advisor for AbbVie, Echosens, Gilead, MSD, Pfizer and Novonordisk.

## Supporting information


**Table S1.** Transition probabilities wherefrom transition matrices has been obtained for building the Markov model.

**Table S2.** Sensitivity analysis strategies evaluating the Markov models’ performance.

**Figure S1.** General structure of the Markov model.
**Figure S2.** Normal and uniform Multivariate Probabilistic Sensitivity Analysis on F2 (A) and F3 (B) fibrosis MASLD patients.

## Data Availability

The authors have nothing to report.
