# Supplementary material for: A Markov Model Unveiling the Impact of Resmetirom on the Natural History of MASLD Patients: A Sistematic Review and Meta‐Analysis
Source: Liver Int. 2025 Mar 11;45(4):e70056. doi: 10.1111/liv.70056 (PMC11894919; doi:10.1111/liv.70056)
Supplement: Supplementary file 1 — Table S1. Transition probabilities wherefrom transition matrices has been obtained for building the Markov model. [file LIV-45-0-s001.docx]

| **Transitions** | **F0** | **F1** | **F2** | **F3** | **CC** | **DC** | **HCC** | **LT** | **LR-M** | **CV-M** | **EHC-M** |
| --- | --- | --- | --- | --- | --- | --- | --- | --- | --- | --- | --- |
| F0 | 0.916055 | 0.041769 | N/A | N/A | N/A | N/A | 0.000411 | N/A | N/A | 0.002361 | 0.0027891 |
| F1 | 0.052737 | 0.8565 | 0.071339 | N/A | N/A | N/A | 0.000385 | N/A | N/A | 0.002213 | 0.0027904 |
| F2 | N/A | 0.068258 | 0.823167 | 0.063401 | N/A | N/A | 0.000388 | N/A | N/A | 0.002459 | 0.0027964 |
| F3 | N/A | N/A | 0.080359 | 0.807339 | 0.070711 | N/A | 0.003619 | N/A | N/A | 0.005733 | 0.0028052 |
| CC | N/A | N/A | N/A | 0.057643 | 0.872944 | 0.044785 | 0.015063 | N/A | N/A | 0.006725 | 0.0028409 |
| DC | N/A | N/A | N/A | N/A | N/A | 0.532373 | 0.036786 | 0.202844 | 0.216958 | 0.00825 | 0.0027883 |
| HCC | N/A | N/A | N/A | N/A | N/A | N/A | 0.351634 | 0.188844 | 0.448062 | 0.008672 | 0.0027883 |
| LT | N/A | N/A | N/A | N/A | N/A | N/A | N/A | 0.892119 | 0.103221 | 0.001872 | 0.0027883 |

**Supplemental Table 1.** Backwards and forwards annual transitions allowed from the models among fibrosis stages, CC, DC, HCC, and death (LR-M, CV-M, EHC-M).

*Abbreviations: CC, compensated cirrhosis, DC, decompensated cirrhosis; HCC, hepatocellular carcinoma; LT, liver transplantation; LR-M, liver-related mortality; CV-M, cardiovascular mortality; EHC-M, extra-hepatic cancer mortality.*
