# Supplementary material for: A Markov Model Unveiling the Impact of Resmetirom on the Natural History of MASLD Patients: A Sistematic Review and Meta‐Analysis
Source: Liver Int. 2025 Mar 11;45(4):e70056. doi: 10.1111/liv.70056 (PMC11894919; doi:10.1111/liv.70056)
Supplement: Supplementary file 3 — Figure S1. General structure of the Markov model. Figure S2. Normal and uniform Multivariate Probabilistic Sensitivity Analysis on F2 (A) and F3 (B) fibrosis MASLD patients. [file LIV-45-0-s002.pptx]

## Slide 1
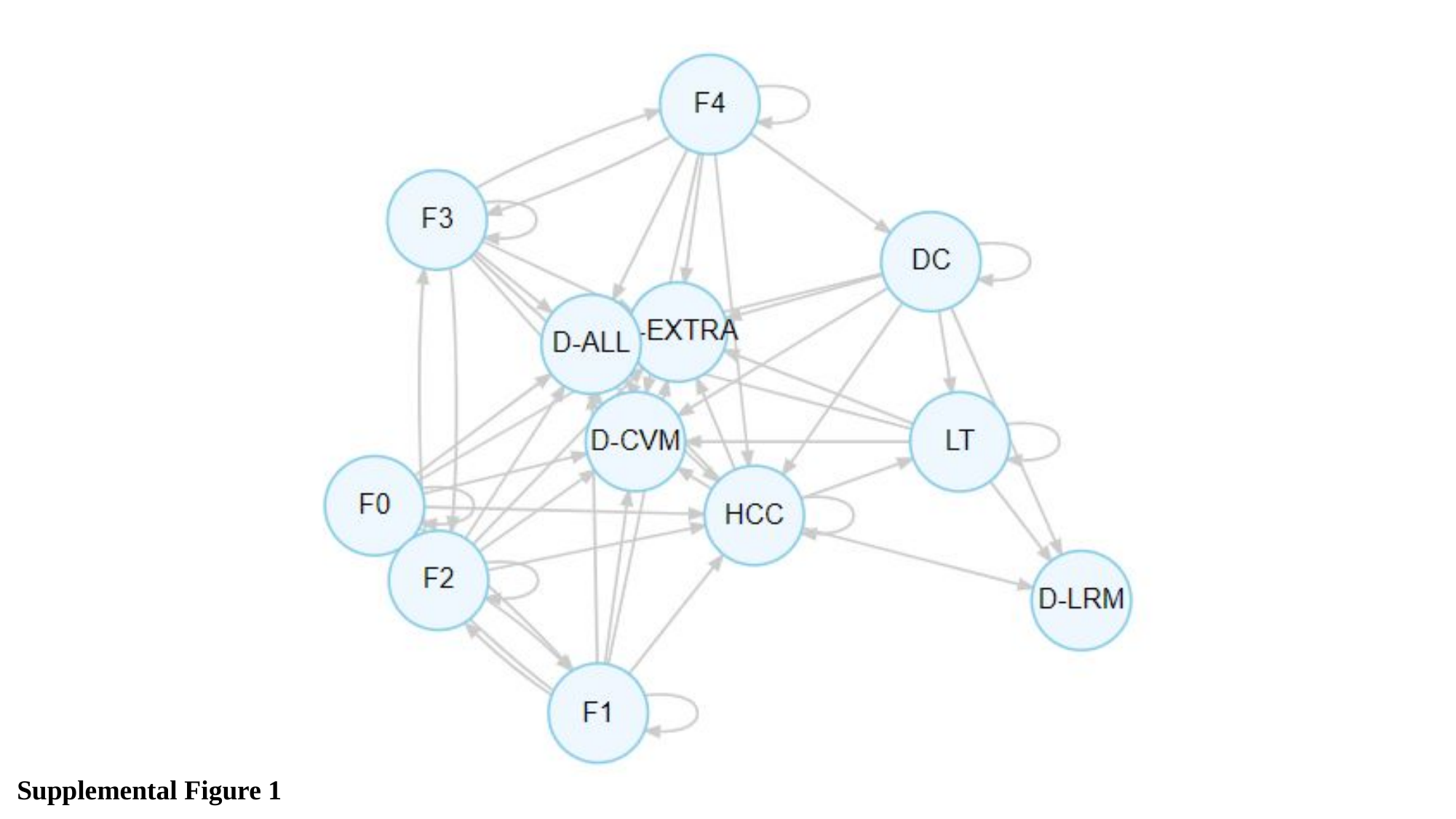

Supplemental Figure 1

## Slide 2
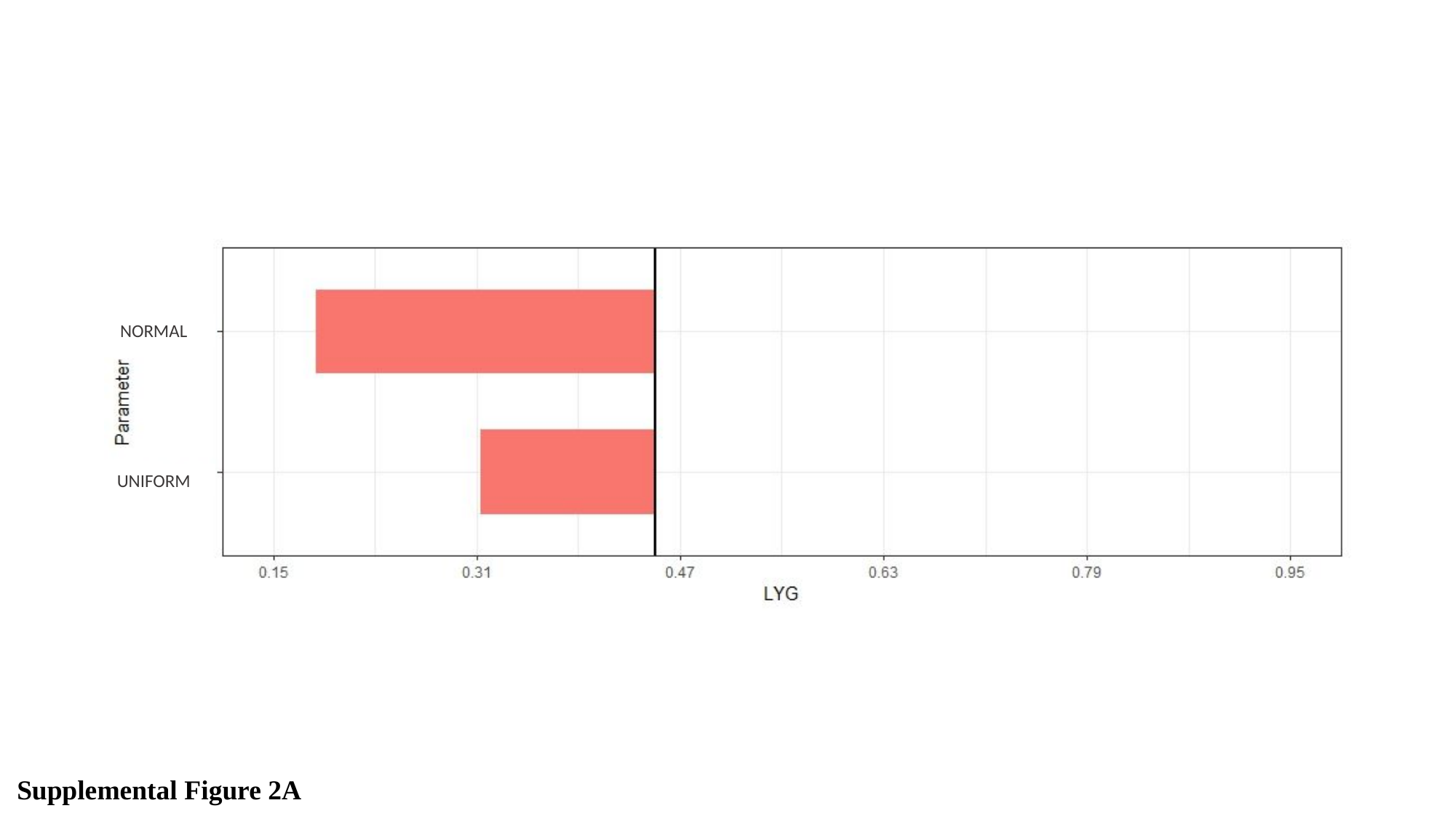

NORMAL
UNIFORM
Supplemental Figure 2A

## Slide 3
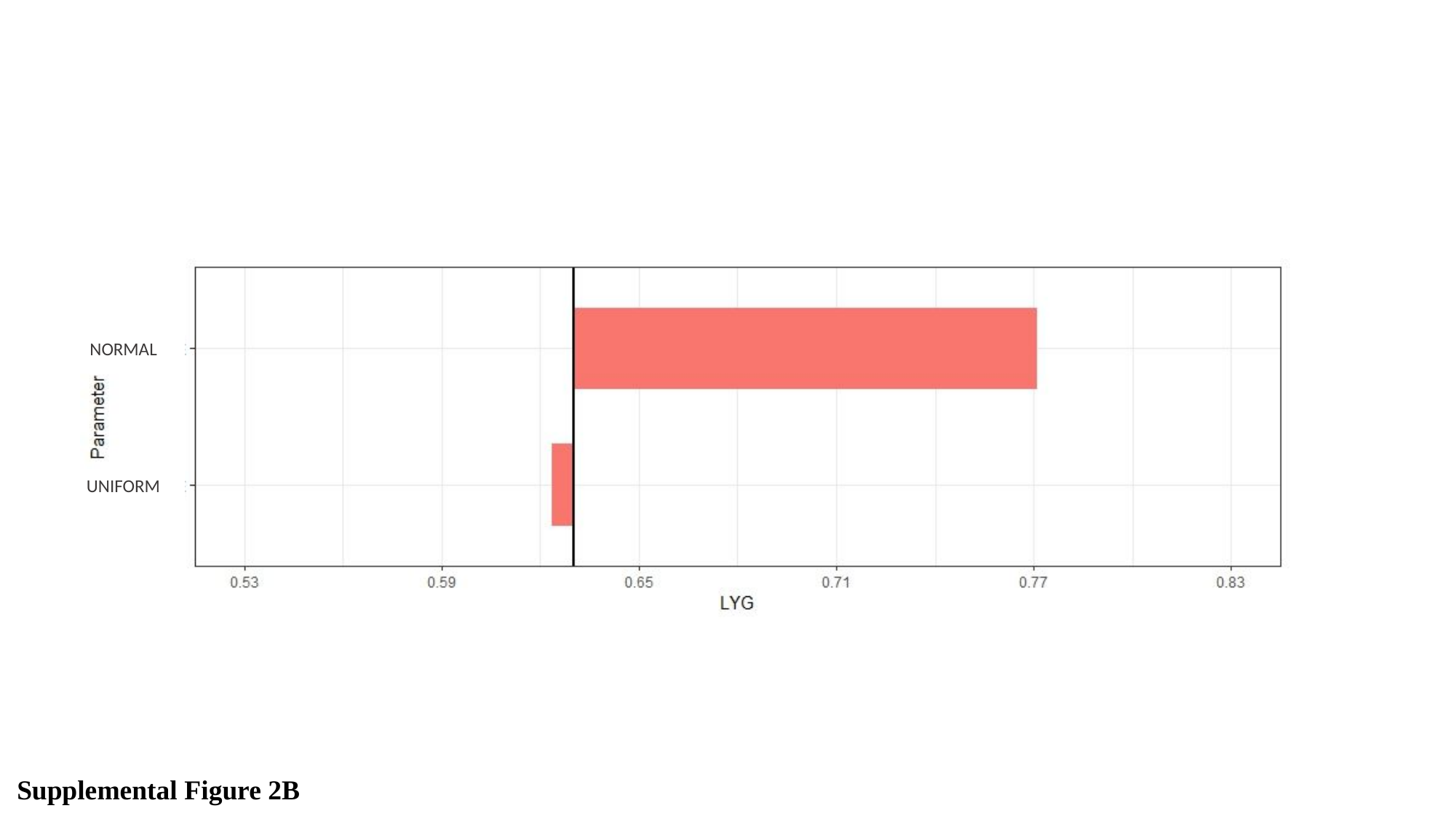

NORMAL
UNIFORM
Supplemental Figure 2B
